# Breast-Milk Microbiota Linked to Celiac Disease Development in Children: A Pilot Study From the PreventCD Cohort

**DOI:** 10.3389/fmicb.2020.01335

**Published:** 2020-06-23

**Authors:** Alfonso Benítez-Páez, Marta Olivares, Hania Szajewska, Małgorzata Pieścik-Lech, Isabel Polanco, Gemma Castillejo, Merce Nuñez, Carmen Ribes-Koninckx, Ilma R. Korponay-Szabó, Sibylle Koletzko, Caroline R. Meijer, M. Luisa Mearin, Yolanda Sanz

**Affiliations:** ^1^Microbial Ecology, Nutrition and Health Research Unit, Institute of Agrochemistry and Food Technology, Spanish National Research Council, Valencia, Spain; ^2^Department of Pediatrics, The Medical University of Warsaw, Warsaw, Poland; ^3^Department of Pediatric Gastroenterology and Nutrition, La Paz University Hospital, Madrid, Spain; ^4^Gluten-Associated Disorder Unit, Institut d’Investigació Sanitària Pere Virgili, Universitat Rovira i Virgili, Reus, Spain; ^5^Pediatric Gastroenterology Unit, La Fe University Hospital, Valencia, Spain; ^6^Celiac Disease Center, Heim Pál Children’s Hospital, Budapest, Hungary; ^7^Department of Pediatrics, Dr. von Hauner Children’s Hospital, University Hospital of Munich, Munich, Germany; ^8^Department of Paediatrics, School of Medicine Collegium Medicum, University of Warmia and Mazury, Olsztyn, Poland; ^9^Department of Pediatrics, Leiden University Medical Center, Leiden, Netherlands

**Keywords:** celiac disease, children, mothers, human milk microbiota, HLA genotype

## Abstract

Celiac disease (CeD) is an immune-mediated disorder triggered by exposure to dietary gluten proteins in genetically predisposed individuals. In addition to the host genome, the microbiome has recently been linked to CeD risk and pathogenesis. To progress in our understanding of the role of breast milk microbiota profiles in CeD, we have analyzed samples from a sub-set of mothers (*n* = 49) included in the PreventCD project, whose children did or did not develop CeD. The results of the microbiota data analysis indicated that neither the BMI, HLA-DQ genotype, the CeD condition nor the gluten-free diet of the mothers could explain the human milk microbiota profiles. Nevertheless, we found that origin country, the offspring’s birth date and, consequently, the milk sampling date influenced the abundance and prevalence of microbes in human milk, undergoing a transition from an anaerobic to a more aerobic microbiota, including potential pathogenic species. Furthermore, certain microbial species were more abundant in milk samples from mothers whose children went on to develop CeD compared to those that remained healthy. These included increases in facultative methylotrophs such as *Methylobacterium komagatae* and *Methylocapsa palsarum* as well as in species such as *Bacteroides vulgatus*, that consumes fucosylated-oligosaccharides present in human milk, and other breast-abscess associated species. Theoretically, these microbiota components could be vertically transmitted from mothers-to-infants during breastfeeding, thereby influencing CeD risk.

## Introduction

Celiac disease (CeD) is an immune-mediated disorder triggered by gliadin proteins and related prolamines (defined as gluten) in genetically susceptible individuals. The disease is characterized by a variable combination of gluten-dependent clinical manifestations, CeD-specific antibodies, HLA-DQ2 or HLA-DQ8 haplotypes and enteropathy (Husby et al., 2012). The HLA-DQ2/8 genes predisposing to CeD and exposure to gluten are necessary but not sufficient to explain CeD onset. Emerging evidence suggests a role for the gut microbiota in CeD risk and pathogenesis (Galipeau et al., 2015; Sanz, 2015; Olivares et al., 2018a, b). The HLA-DQ2/8 genotype is associated with alterations in the early colonization pattern of infants at family risk of developing CeD (Palma et al., 2012; Olivares et al., 2015b). Early feeding patterns, including the type of milk-feeding (breastfeeding or formula) and the introduction of gluten into the infant’s diet are considered determinants of CeD onset (Silano et al., 2016). Both milk-feeding practices, particularly breastfeeding (Palma et al., 2012), and the amount of gluten in the diet (Sanz, 2010; Hansen et al., 2018) influence the gut microbiota composition and could, thus, act as moderators of CeD risk. Yet, little is known about how the host’s genetics and the environment interact with the gut microbiota, possibly contributing to the chain of causative mechanisms leading to CeD.

The first retrospective observational studies, which included control individuals from a general population, found a preventive effect of breastfeeding against later CeD development, particularly when breastfeeding was maintained parallel to introducing gluten into the infant diet (Auricchio et al., 1983; Stevens et al., 1987; Greco et al., 1988). Since then, several prospective and interventional studies including children with genetic risk predisposing to CeD or diabetes mellitus type I, have also tested this hypothesis but conclude that breastfeeding and its duration do not influence CeD risk (Szajewska et al., 2015; Silano et al., 2016). Some prospective studies report there is no association between CeD risk and gastrointestinal infections (Szajewska et al., 2015; Ziberna et al., 2016), however, our prospective gut microbiota analysis found an increased prevalence of pathogenic bacteria (enterotoxigenic *Escherichia coli*) in the feces of children with increased risk of developing CeD (Olivares et al., 2018a). Furthermore, findings from the prospective study TEDDY indicate that gastrointestinal infections increase the risk of CeD autoimmunity, and that the risk depends on other factors such as breastfeeding, the HLA genotype, infant gluten consumption, and rotavirus vaccination (Kemppainen et al., 2017). Therefore, the fact that previous prospective studies failed to control all interacting elements may well explain the inconsistency regarding the role of environmental variables like breastfeeding, which influence both gut microbiota and oral tolerance to allergens/gluten, in CeD onset.

A few studies have also suggested that breast-milk composition differs depending on the mother’s health status, which may influence its potential protective effect in CeD and partially explain the discrepant findings across studies. In particular, breast milk from control mothers and mothers with CeD was found to differ in microbiota composition (bifidobacteria) and immune mediators [secretory immunoglobulin A (sIgA) and transforming growth factor (TGFβ1)] and total IgA, which could be involved in the development of oral tolerance to gluten (Olivares et al., 2015a; Roca et al., 2018). However, this hypothesis has not been directly proven so far. Moreover, differences in mothers’ milk microbiota according to their CeD condition could lead to differences in the bacteria transmitted through breastfeeding and colonizing the infants’ gut, which could theoretically affect early immune development and disease risk (Olivares et al., 2015a; Demmelmair et al., 2020).

To progress in our understanding, the present pilot study aims to investigate the relationship between mother’s breast milk microbiota profiles and CeD development in their offspring, including the analysis of potential covariates. A strict methodology was applied to control for cross-contamination and accurately identify human-milk associated bacteria.

## Materials and Methods

### Study Participants and Sample Collection

The PreventCD cohort consists of 944 children with at least one first-degree relative with CeD and available genotype HLA-DQ2 or HLA-DQ8 enrolled at birth, between 2007 and 2010, in Croatia, Germany, Hungary, Israel, Italy, Netherlands, Poland, and Spain. The PreventCD project (prevention of celiac disease^1^) investigates the influence of genetic, immunologic and environmental factors on the risk of developing CeD. All the children were assessed regularly from birth onward for CeD development (monthly until the age of 1 year, bimonthly until the age of 2 years, and trimonthly onward). We periodically monitored parent-reported health status, anthropometrics, gluten consumption and specific CeD serology [IgA against tissue-transglutaminase (TGA)] as previously reported (Vriezinga et al., 2014). The parents of children with elevated TGA and/or CeD-associated symptoms suggestive of CeD, were offered small bowel biopsies to confirm the diagnosis or were diagnosed without biopsies according to the criteria of the European Society for Paediatric Gastroenterology Hepatology and Nutrition (ESPGHAN) (Husby et al., 2012). The disease onset in children of mothers providing CeD milk samples was diagnosed at 41.2 ± 6.38 months after birth (95% CI 34.8–47.6). Maternal milk samples were collected at 9 months after birth. Mothers expressed their milk manually or with a pump, but the preferred way for the collection was not recorded. No further specifications pre- or post-milk sampling or time of day were given to the mothers. The milk samples were frozen at −20°C at home, transferred to the hospital on ice to avoid thawing and stored at −80°C until processed for microbiota analyses. A total of 49 breast-milk samples from the mothers of the Dutch, Polish, Spanish, Hungarian, and German participants in PreventCD stored at the Leiden University Medical Center were used in this assessment. They corresponded to milk from mothers of 25 children who developed CeD and from mothers of 24 children who did not develop CeD, referred to as “controls.” The study was registered at ISRCTN (ISRCTN74582487) on February 26th, 2007. The first and last children were included between 26th of May 2007 and the 25th of September 2010, respectively. This study was conducted according to the guidelines laid down in the Declaration of Helsinki and it was approved by all medical ethics committees of the participating centers.

### DNA Isolation and Processing

Approximately 9 mL of milk sample was combined with 1 mL sterile 10× PBS + 0.1% Tween 20 (v/v) and mixed gently. The samples were then filtered through 5.0 μm membranes (Millipore) to eliminate eukaryotic cells and the flow-through was recovered for further processing. Filtered samples were centrifuged at 10,000 *g* for 10 min and pellets were stored in 1.5 mL nuclease-free tubes until processing for DNA extraction. Cell pellets were used for DNA isolation using the PowerFecal^®^ DNA Isolation Kit (Mo Bio Laboratories) following manufacturer instructions and with a prior lysis treatment to improve DNA extraction from resistant species. Thus, pellets were resuspended in 300 μL sterile PBS, containing 20 U mutanolysin (Sigma) and 250 μg lysozyme (Sigma) and incubated during 60 min at 37°C. Given the potentially low bacterial load present in human milk samples, we included three negative controls corresponding to the different DNA extraction batches. These controls included a 1 mL aliquot of the 10× PBS + 0.1% Tween 20 buffer used to homogenized milk samples prior filtration and DNA extraction. These negative controls were equally processed during DNA extraction, PCR, and amplicon sequencing. The human-milk-derived genomic DNA was quantified by fluorescence-based methods such as Qubit 3.0 and the Qubit dsDNA HS Assay Kit (Thermo Fisher Scientific, Waltham, MA, United States), and 1 μL DNA (0.5–5 ng) aliquot was used for microbiota analysis. The V4–V5 hypervariable regions of the bacterial 16S rRNA gene were amplified by 25-PCR cycles, including the following stages: 95°C for 20 s, 40°C for 30 s, and 72°C for 20 s. For PCR, Phusion High-Fidelity Taq Polymerase (Thermo Fisher Scientific) and the 6-mer barcoded primers S-D-Bact-0563-a-S-15 (AYTGGGYDTAAAGNG) and S-D-Bact-0907-a-A-20 (CCGTCAATTYMTTTRAGTTT), were used to targeting the bacterial 16S rRNA gene (Klindworth et al., 2012).

### Sequencing and Data Processing

Dual barcoded PCR products, consisting of ∼400 bp, were purified from triplicate reactions by using the Illustra GFX PCR DNA and Gel Band Purification Kit (GE Healthcare) and quantified through Qubit 3.0 and the Qubit dsDNA HS Assay Kit (Thermo Fisher Scientific, Waltham, MA, United States). Samples were multiplexed by combining equimolar quantities of amplicon DNA (100 ng per sample) and sequenced in one lane of Illumina MiSeq platform (Eurofins Genomics GmbH, Ebersberg, Germany) with 2 × 300 PE configuration. The volume of purified PCR from negative controls (with no signal of amplified DNA) to be combined in the sequencing library was calculated as the median of the volume used for the entire set of samples (∼6 μL). Raw data were delivered in fastq files and paired-ends with quality filtering were assembled using *Flash* software (Magoc and Salzberg, 2011). Sample de-multiplexing was carried out using sequence information from respective DNA barcodes and *Mothur v1.36.1* suite of analysis (Schloss et al., 2009). After assembly and barcodes/primers removal, the sequences were processed for chimera removal using *Uchime* algorithm (Edgar et al., 2011) and SILVA reference set of 16S rRNA gene sequences (Release 110) (Quast et al., 2013). Taxonomy assessment was performed using the RDP classifier v2.12 (Wang et al., 2007), using a high-quality and normalized subset of 5,000 sequences per sample, randomly selected after shuffling (10,000×) of the chimera removed dataset. The operational taxonomic unit (OTU)-picking approach was performed with the normalized subset of 5,000 sequences and the *uclust* algorithm (97% sequence identity) implemented in USEARCH v8.0.1623 (Edgar, 2010) discarding singletons and doubletons during the OTU clustering. Non-parametric linear discriminant analysis (LDA) among cases and controls was performed to measure differences among human milk microbial communities at different taxonomy level (Segata et al., 2011). Taxonomy identity of OTUs with differential abundance was assessed using a Blast-based approach and the NCBI non-redundant 16S rRNA gene database as well as the SINA aligner and SILVA database annotation (Pruesse et al., 2012). Common descriptors of the alpha diversity such as Chao’s index, observed OTUs, Shannon index, reciprocal Simpson’s index, dominance index, and phylogenetic distance were assessed using the OTU level information and the *qiime* v1.9.1 suite of analysis (Caporaso et al., 2010).

### Potential Reagent Contaminants Identification and Removal

Given the very low bacterial load present in human milk, data analysis could be biased by the presence of foreign DNA coming from DNA extraction reagents, PCR, and sequencing approaches. To reduce this noise, we included negative controls that enable tracing of the bacterial species unpredictably present in reagents. In this way, we retrieved a few hundred DNA reads from respective negative controls, and identified dozens of bacterial groups present in those controls. The negative controls exhibited quite a different microbiota profiling compared to samples using multivariate analysis (Supplementary Figure S1). An abundance-based estimation of likely contaminant OTUs was performed by assessing predominant OTUs of negative control samples (>0.1% in average). Then, those OTUs were removed from control and cases samples if the abundance median was less than five reads (0.1%).

### Statistical Analysis

The statistical assessment of individual parameters and comparison among groups was performed with Wilcoxon rank-sum test or *t-test* (Welch’s correction) according to Shapiro–Wilk normality test results, all the above implemented in R v 3.5^1^. *Qiime* v1.9.1 was also used to analyze the global structure of microbial communities (beta diversity) through weighted and unweighted unifrac metrics as well as to perform principal coordinate analysis (PCoA) and statistical assessment with permutation-based test (Permanova) (Caporaso et al., 2010). Non-parametric correlations (Spearman’s *rho*) between principal coordinate (PC) values and OTU abundances were assessed and corrected for multiple comparisons, using the false discovery rate (FDR) *post hoc* test. Linear mixed models (LMM) were also used with log-transformed OTUs and clinical metadata to determine potential covariates influencing the human milk microbiota (hMM) by using the *nlme* R package. The metadata used to establish associations with hMM profiles include both children’s covariates (*disease*, *gender*, HLA-DQ *genotype, birth year*, and *birth season*) and mother’s covariates (*disease*, HLA-DQ *genotype, birth year, maternity age, gluten-free diet, delivery mode, BMI, origin country*). CeD genetic risk based on HLA-DQ genotyping (scale from 1 to 5) was calculated according to Vriezinga et al. (2014) for the PreventCD cohort, the lower the score the higher the DQ-associated genetic risk of CeD. Graphics and plots were designed in R v 3.5 and *ggplot2* package^2^.

## Results

### Demographic and Clinical Characteristics

Summarized in Table 1 are the demographic and clinical variables taken into account in the present study, due to their potential relationship with CeD risk and the microbiota. We found no significant difference in distribution of such variables between the two groups compared (controls and CeD cases).

**TABLE 1:** Demographic and clinical variables of the mothers and children who developed celiac disease (CeD) and who did not (controls).

	**Control group^1^ (*N* = 24)**	**CeD group^1^ (*N* = 25)**	**Statistics^2^**
Maternity age	32.4 ± 2.9	33.5 ± 2.6	*t* = 1.338, *p* = 0.188
Offspring sex	Female = 13	Female = 14	χ^2^ = 0.000, *p* = 1.000
	Male = 11	Male = 11	
Mother with CeD	Yes = 14	Yes = 13	χ^2^ = 0.025, *p* = 0.874
	No = 10	No = 12	
Mother following GFD	Yes = 12	Yes = 11	χ^2^ = 0.018, *p* = 0.893
	No = 12	No = 14	
Mother CeD risk^3^	3.04 ± 1.60	2.75 ± 1.36	*W* = 256, *p* = 0.491
Offspring CeD risk^3^	2.66 ± 1.24	2.44 ± 1.19	*W* = 271, *p* = 0.538
Offspring birth year	2007 = 6	2007 = 7	χ^2^ = 3.173, *p* = 0.366
	2008 = 15	2008 = 11	
	2009 = 3	2009 = 5	
	2010 = 0	2010 = 2	
Delivery mode^4^	Vaginal = 19	Vaginal = 18	χ^2^ = 2.001, *p* = 0.367
	C-section = 0	C-section = 2	
	NA = 5	NA = 5	
Maternal BMI^4,5^	19.2 ± 1.9	19.0 ± 1.2	*W* = 78, *p* = 0.751
Origin country	Germany = 8	Germany = 9	χ^2^ = 0.206, *p* = 0.995
	Hungary = 7	Hungary = 6	
	Netherlands = 3	Netherlands = 3	
	Poland = 1	Poland = 1	
	Spain = 5	Spain = 6	

*^1^Continuous and discrete variables are shown as the average of the distribution ± standard deviation (SD). ^2^Statistical comparison of variables between groups was assessed according to their nature and distribution. Categorical variables were evaluated with Chi-square test with Yates’ continuity correction, whereas differences in continuous and discrete variables were evaluated with *t*-test (Welch’s correction) and Wilcoxon rank-sum test for unpaired samples according to the normal distribution of variables, tested with the Shapiro–Wilk normality test ahead. ^3^CeD genetic risk based on HLA-DQ genotyping. Scores (DQ risk group) were calculated according to Vriezinga et al. (2014) for the PreventCD cohort. The lower the score the higher the DQ-associated genetic risk of CeD. ^4^Partial data recorded. Metadata for some samples is missing. ^5^Weight and height recorded post-partum at milk sampling time. BMI, body mass index; GFD, gluten-free diet; NA, not available metadata.*

### Alpha and Beta Diversities

**FIGURE 1:**
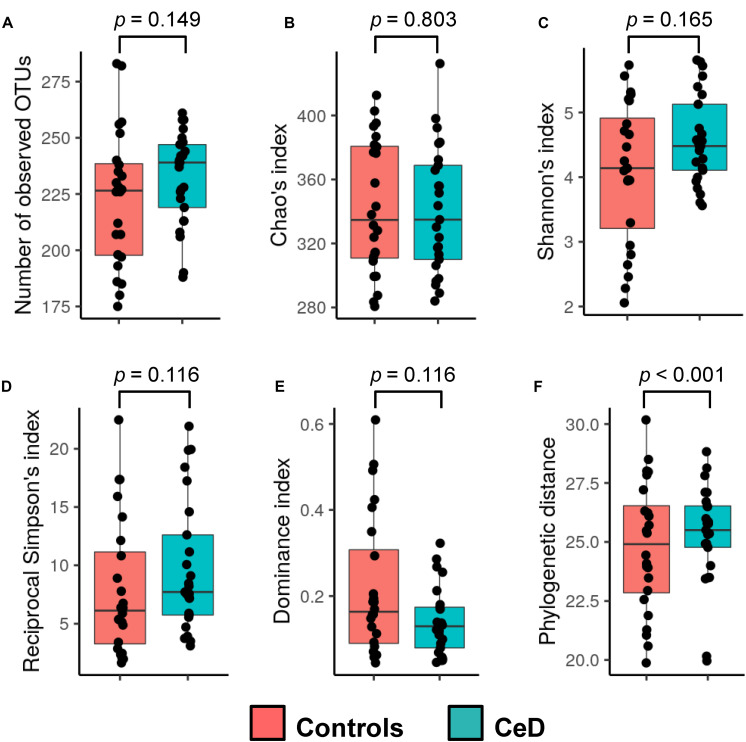
Alpha diversity of the human milk microbiota. The observed richness **(A)**, Chao’s index **(B)**, Shannon’s index **(C)**, reciprocal Simpson’s index **(D)**, dominance index **(E)**, and phylogenetic distance **(F)** were calculated for samples and compared between the study groups (cases and controls; see legend color). Non-parametric Wilcoxon rank-sum test (unpaired samples) was used to calculate differences among groups. The *p*-values resulting from the statistical assessment are shown.

Six common ecological descriptors were employed to assess the alpha diversity of the hMM in samples obtained from mothers enrolled in the PreventCD project included in this study. Globally, we found no differences in richness, entropy, dominance, or diversity in control and CeD-positive cases (mothers whose children later developed CeD), but we did retrieve an augmented phylogenetic distance among OTUs present in CeD samples (*p* < 0.001) (Figure 1). Regarding beta diversity, weighted and unweighted Unifrac metrics were calculated and a Permanova test was used to identify differences in hMM structure as a function of the different variables considered (see Table 1). As expected, we found that hMM profiles were influenced primarily by mothers’ origin country (unweighted unifrac based Permanova test = 1.53, *p* = 0.001, weighted unifrac based Permanova test = 3.20, *p* = 0.001) (Supplementary Figure S2A); however, we found that offspring’s year of birth, referred to henceforth as “offspring birth year” (unweighted unifrac based Permanova test = 1.40, *p* = 0.001, weighted unifrac based Permanova test = 1.66, *p* = 0.069) also resembled to shape the hMM (Supplementary Figure S2B). No other covariates expected to theoretically affect hMM, such as maternal BMI and delivery mode, were detected. Applying an exploratory multivariate PCoA, we found that distribution of the PC1 values of weighted unifrac correlates with birth year, suggesting that offspring birth year would be a covariate of hMM, likely influencing its composition. Additionally, we observed a gradient-like distribution of PC1 values across all samples dependent on children’s birth year (Supplementary Figure S2B).

To discover the OTU abundance correlated with the PC1 values of weighted unifrac metrics, the Spearman’s *rho* parameter was calculated between the PC1 values and the OTUs, and the *p*-values obtained were corrected by multiple testing (FDR ≤ 0.05). As a result, we revealed eighteen OTUs (*rho* ≥ 0.60, *p* < 0.001) positively correlated with PC1 values indicating that such phylotypes tended to be more abundant in breast milk from mothers delivering their children during 2007 (with higher PC1 values) and reduced in the breast milk of mothers delivering their offspring later on (2010). This could also imply a reduction in the delivery of these phylotypes into the intestine of infants during breastfeeding throughout the study. When a Blast-based search was performed in order to unveil the bacterial identity of such DNA sequences, we found conclusive taxonomy identification for 13 out of 18 OTUs. In particular, the abundance of the following species appear to be in higher proportions in the breast-milk of mothers involved earlier in this study: *Anaerostipes hadrus* (OTU41), *Alistipes shahii* (OTU39), *Parabacteroides golsteinii* (OTU64), *Prevotella copri* (OTU7), *Faecalibacterium* spp. (OTU91), *Bacteroides uniformis* (OTU27), *Bacteroides xylanisolvens* (OTU2), *Alistipes putredinis* (OTU142), *Butyrivibrio crossotus* (OTU40), *Alistipes obesi* (OTU10), *Parabacteroides johnsonii* (OTU135), *Bifidobacterium bifidum* (OTU32), and *Paraprevotella clara* (OTU101). Conversely, negative correlations between PC1 values and OTU abundances (*rho* ≤ 0.56, *p* < 0.001) of the species *Bacillus wiedmannii* (OTU33), *Enterococcus saigonensis* (OTU197), and several species of the *Staphylococcus* genus (OTU193, OTU331, OTU84, and OTU2) were found. This indicates that the abundance of these bacterial species increased progressively in breast milk throughout the study.

### Bacterial Taxonomic Composition of hMM

**FIGURE 2:**
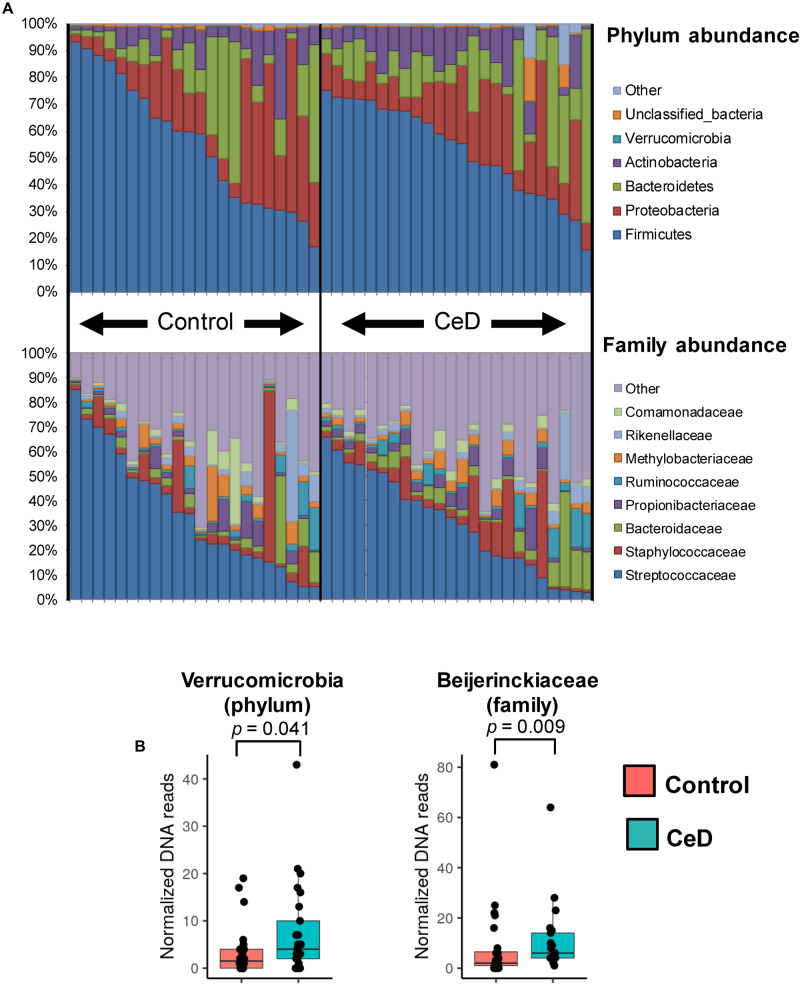
Bacterial phyla and families distribution in human milk microbiota. **(A)** The phylum (top bar plot) and family (bottom bar plot) distribution in the whole set of samples analyzed show the most predominant members present in different human milk samples. **(B)** Taxonomic categories with differential abundance in the study groups (cases and controls). The distribution of DNA reads assigned to Verrucomicrobia phylum and Beijerinckiaceae family are presented in boxplots. In both cases the abundance of reads is higher in case samples (CeD developed in the offspring) than in controls (*p* = 0.041 and *p* = 0.009, respectively).

The inventory of the bacterial phyla and families present in breast milk samples was evaluated by assigning the respective taxonomy categories to DNA reads with the RDP classifier v2.12 as shown in Figure 2A. We found that Firmicutes and Proteobacteria were the most common bacterial phyla present in the entire set of samples followed by Bacteroidetes and Actinobacteria. In terms of family distribution, we observed that members of the Streptococcaceae and Staphylococcaceae families were predominantly present in human milk samples. Proteobacteria was mainly represented by members of Methylobacteriaceae and Comamonadaceae families, whereas Actinobacteria and Bacteroidetes were essentially represented by Propionibacteriaceae and Bacteroidaceae families, respectively. At first glance, there was a high inter-individual variability in the microbial composition of the samples and no abundance patterns could be visually associated with the human milk control and CeD samples. Applying a LDA we found that milk of mothers whose children later developed CeD contained a high proportion of Verrucomicrobia (LDA score = 3.18, *p* = 0.041) and *Beijerinckiaceae* species (LDA score = 3.36, *p* = 0.009) (Figure 2B). We also detected some differences in the abundance of specific OTUs in control mothers and mothers whose infants developed CeD, being OTU13 (*Bacteroides vulgatus*), OTU42 (*Methylobacterium komagatae*), OTU63 (*Parabacteroides distasonis*), and OTU54 (*Methylocapsa palsarum*) (Table 2).

**TABLE 2:** OTUs with differential abundance in breast milk samples from mothers whose children later develop celiac disease (CeD) compared to those whose children remained healthy (controls).

**OTU**	**Association^1^**	**LDA score**	***p*-Value**	**Blast (% alignment, % identity)^2^**
OTU13	CeD	3.67	0.0324	*Bacteroides vulgatus* (100, 99)
OTU42	CeD	2.87	0.0147	*Methylobacterium komagatae* (100, 99)
OTU63	CeD	2.81	0.0394	*Parabacteroides distasonis* (100, 100)
OTU54	CeD	2.70	0.0121	*Methylocapsa palsarum* (100, 98)

*^1^Association with CeD development; the breast milk samples showed increased abundance in the taxonomic group recorded. ^2^Taxonomic identification based on best hit during the alignments using the non-redundant 16S NCBI database.*

### Associations Between the hMM and Different Covariates

A LMM analysis was performed to evaluate the possible influence of other clinical covariates on the hMM profiles. As previously detected by beta diversity analysis, this additional data analysis indicated that hMM structure was influenced only by the mothers’ origin country and offspring birth year, explaining approximately 52 and 11% of observed variability, respectively. The above was inferred from the observation that 71 OTU categories (*p* < 0.05), of the 247 most abundant identified (>0.01% of abundance), appeared to be differentially distributed across the offspring birth period between 2007 and 2010, whereas 93 OTUs appeared with differential abundance among the five origin countries (*p* < 0.05). The OTU categories and their most probable taxonomic identification with larger negative and positive associations according to offspring birth year are shown in Supplementary Figure S3. Of these, we highlight *Pseudomonas* spp. (OTU14) and *Acinetobacter* spp. (OTU22) (both from Pseudomonadales order), whose abundance increased progressively with offspring birth year irrespective of the origin country of samples (set as random effect on LMM). The remaining covariates contributed to explain the hMM profile to a lesser extent. Moreover, we identified 8 OTUs differentially present in the two groups of mothers, i.e., those whose children later developed CeD and those whose children remained healthy, after adjusting for all other covariates (Table 3). When comparing LDA and LMM data, we found four other OTUs associated with the microbiota of mothers whose infants developed CeD and two of them certainly correlated with specific species, including *Peptoniphilus timonensis* (OTU62) and *Actinomyces turicencis* (OTU483).

**TABLE 3:** OTUs with differential abundance in breast milk samples from mothers whose children later developed celiac disease (CeD) compared to those whose children remained healthy (controls) according to LMM analysis.

**OTU**	**Association^1^**	**Variation^2^**	***p*-Value**	**Blast (% alignment, % identity)^3^ SINA classsification^4^**
OTU179	CeD	−1.03	0.0010	(g) *Muribaculum* sp. (100, 95)
				(f) Muribaculaceae (92.8)
OTU62	CeD	−1.09	0.0195	(s) *Peptoniphilus timonensis* (100, 99)
				(g) *Peptoniphilus* (99.4)
OTU42	CeD	−1.12	0.0193	(s) *Methylobacterium komagatae* (100, 99)
				(g) *Methylobacterium* (99.4)
OTU54	CeD	−1.04	0.0233	(s) *Methylocapsa palsarum* (100, 98)
				(f) Beijerinckiaceae (97.9)
OTU13	CeD	−1.19	0.0320	(s) *Bacteroides vulgatus* (100, 99)
				(g) Bacteroides (99.7)
OTU143	CeD	−0.77	0.0257	(s) *Atopostipes suicloacalis* (100, 97)
				(f) Carnobacteriaceae (100)
OTU483	CeD	−0.60	0.0343	(s) *Actinomyces turicensis* (100, 99)
				(g) *Actinomyces* (99.7)
OTU73	CeD	−0.85	0.0317	(s) *Wautersiella falsenii* (100, 99)
				(g) *Empedobacter* (98.5)

*^1^Association with celiac disease (CeD) development; the breast milk samples showed increased abundance in the taxonomic group recorded. ^2^Variation calculated taking into account the control samples as reference and based on normalized DNA read counts by logarithmic transformation. ^3^Taxonomic identification based on best hit during the alignment using the non-redundant 16S rRNA gene NCBI database. ^4^Taxonomic identification based on SINA aligner using the SILVA reference database and sequence identity value against the last common ancestor (lca). S, species; g, genus; f, family.*

## Discussion

Many recent hMM surveys report the microbiota profiles potentially involved in health and disease, not only of mothers but also of their offspring (Sakwinska and Bosco, 2019). Controversy exists on the most suitable methodology to accurately analyze the microorganisms present in human milk since this niche has a very low bacterial load. This makes it prone to misidentification of microbial profiles due to cross-contaminations of samples with foreign DNA from different processing stages and reagents used for DNA isolation, PCR amplification and sequencing (Salter et al., 2014). Therefore, the present study aimed to control contaminant DNA from reagents to minimize this source of bias when assessing the microbial signatures of different human milk samples (Sakwinska and Bosco, 2019), and to link specific microbial features with the development of CeD in children.

We found that the microbiota of CeD samples was more diverse than that of controls given the differences reflected in the phylogenetic distance metrics. Moreover, most of the primary variables and factors thought to modulate the hMM (genetic risk, CeD mother condition, mothers’ gluten-free diet, mothers’ age, delivery mode, and mothers’ BMI) exhibited no significant impact on its structure. Conversely, and apart from the well-known geographical influence on human microbiota assessments (Kumar et al., 2016; Deschasaux et al., 2018; He et al., 2018), replicated in this study, we found that hMM was primarily influenced by offspring birth year in our study population. As shown, human milk samples from 2007 to 2008 contained a higher abundance of strictly anaerobic and gut-associated microbes such as *Anaerostipes* spp., *Parabacteroides* spp., *P. copri, Faecalibacterium* spp., *Bacteroides* spp., *Alistipes* spp., and *Bifidobacterium* spp. among others, whereas later samples (2009–2010) were enriched in *Bacillus*, *Enterococcus*, and *Staphylococcus* species. Therefore, the strictly anaerobic bacteria found in the samples first collected seemed to be progressively replaced by aerobic or facultative bacteria in the samples of mothers collected afterward. This could be due to sample storage time and freeze-thaw cycles but, in our study, the samples stored for longer contained a higher abundance of strictly anaerobic bacteria. Therefore, we hypothesize that this shift might be the result of changes in lifestyle and environmental variables, which shaped the assembly of such bacterial communities in mothers’ milk. Moreover, given that abundance of any of the OTUs outlined above was not influenced by the mothers’ origin country, delivery mode, mothers’ BMI or mothers’ disease condition and gluten-free diet followed, the changes associated with the offspring birth year retrieved from this sample cohort of multi-national origin might be suggestive of a short-term switch of hMM signatures across the population. The above idea would be possible because of the lifestyles and environmental factors in urban settlements has been already associated with worsening human health, including human milk quality (Giles-Corti et al., 2016; Pajewska-Szmyt et al., 2019), and theoretically these factors could also negatively impact the breast milk microbiota. Nonetheless, we are conscious that the inspection of a greater sample size across a considerably longer sampling period will be mandatory to test such a hypothesis. Alternatively, we cannot discard that this hMM profile associated with offspring birth year may be a direct consequence of milk expression method for infant feeding, which has been demonstrated to induce meaningful changes in the human milk by reducing diversity and increasing potential pathogens (Moossavi et al., 2019). Despite the fact that our metadata lacks direct information of the milk expression method used, this hypothesis is plausible since there is an increased tendency to use expressed breast milk feeding as routine practice in developed countries, as those included in our study (reviewed by Johns et al., 2013).

Analyses of the taxonomic features present in hMM at the phylum and family level indicate that Streptococcaceae and Staphylococcaceae species are predominant members of the hMM. These data are consistent with previous inventories of microbial species identified in human milk, where the predominance of Proteobacteria and Firmicutes seems to be a common feature (Biagi et al., 2017; Li et al., 2017; Murphy et al., 2017). Moreover, presence of certain bacterial groups associated with skin microbiota (like Propionibacteriaceae species) also appears to be frequent in this type of sample.

We found increased abundance of *B. vulgatus* in breast-milk samples of mothers whose children developed CeD. This species is capable of efficiently fermenting fucosylated oligosaccharides present in human milk (Marcobal et al., 2010) and, therefore, human milk oligosaccharides could support their prevalence in breast milk samples. Furthermore, *B. vulgatus* has been previously linked to CeD as infants with a high genetic risk showed an increased prevalence of this genus (Sanchez et al., 2011). Moreover, *P. distasonis* was also present in higher proportions in such samples. Although there are no previous reports identifying the presence of these common human gut microbes in breast milk, the abundance of *B. vulgatus* has been associated with irritable bowel disease (IBD) severity (Schirmer et al., 2018), and that of *P. distasonis* with Crohn’s disease (Lopetuso et al., 2018). This evidence could hint as to the potentially harmful effects of the transmission of these bacterial species from mothers to their infants. Future studies to unveil potential relationships between human milk oligosaccharides and the abundance of microbial species found in hMM associated with CeD children should be warranted. This would provide further insights on the mother-child vertical microbiota transmission since the abundance of some species in fecal microbiota of breast-fed infants seem to correlate to oligosaccharides present in human milk (Wang et al., 2015). Consequently, precise mechanistic studies are necessary to test such hypothetical associations.

Interestingly, we also found environmental-associated microbes to be present in human milk, showing differential abundance between study groups with higher abundance in CeD samples. This included, in particular, *Methylobacterium* species of the Rhizobiales order (phylum Proteobacteria), which are commonly known as one-carbon compound reducer microorganisms widely found in soil and plant surfaces. Moreover, species of the *Methylocapsa* genera, members of the Beijerinckiaceae family, formed part of the hMM after minimizing the confounding effects of contaminant DNA coming from reagents. Species belonging to the *Methylobacterium* genus are prevalent in water pipes and reservoirs (Barbeau et al., 1996; Vaz-Moreira et al., 2017) and as contaminants of industrial milk samples (Bracke et al., 2014). In addition, some studies also indicate that *Methylobacterium* species are part of the human oral and skin microbiota (Hung et al., 2011; Alekseyenko et al., 2013) and, therefore, their presence in breast milk microbiota could partly be due to their transmission via the infant’s oral cavity, an event that has been recently hypothesized as important modulator of the hMM (Moossavi and Azad, 2019; Moossavi et al., 2019). Interestingly, a recent microbiota analysis of human breast tissue through 16S rRNA gene amplicon sequencing has also reported the presence of *Methylobacterium* (among other environmental microbes such as *Ralstonia*) in healthy and cancerous breast biopsies (Costantini et al., 2018), supporting the presence of these type of microbes in human breast-related samples. Although the presence of such methylotrophs in hMM is corroborated by our study, their particular role in CeD onset needs further evaluation. In this regard, recent reports of the ability of *Methylobacterium* species to utilize organic acids and ethanolamines, and to produce central metabolic compounds should be investigated further to understand their possible functional role in health and disease (Yang et al., 2009).

A LMM-based analysis to control several covariates of the microbiota enabled us to distinguish additional microbial species potentially associated with the development of CeD in children due to its increased abundance in the milk of their respective mothers. As a result and, in addition to those species described in Table 2, we found that the abundance of *P. timonensis* and *Actinomyces turicensis* related OTUs were increased in milk samples from mothers whose children developed CeD. In this regard, *Peptoniphilus* species have been considered as potential inducers of pro-inflammatory signals associated with an increased risk of suffering from human immunodeficiency virus (HIV) infection and wound severity in skin ulcers (Liu et al., 2017; Park et al., 2019), which could also confer higher susceptibility to other inflammatory conditions such as CeD. Moreover, breast abscesses caused by *A. turicensis* and *Peptoniphilus harei* also indicate these bacterial taxa are potentially pathogenic (Le Bihan et al., 2019). On the other hand, the LMM analysis also revealed several species potentially associated with offspring birth year, which could play an important role in the colonization of the infant’s gut during breastfeeding. We found that *P. copri*, a species recurrently associated with traditional lifestyle and non-Westernized populations (Fragiadakis et al., 2019), was more abundant in milk samples of mothers who delivered their children in 2007, regardless their origin’s country, BMI, and mothers’ disease status, and the abundance seemed to decline in samples from mothers who delivered their children in subsequent years. Interestingly, a similar pattern of evolution has been found for the abundance of *Prevotella* species, which seems to have been reduced in the human gut microbiota, particularly as a result of the transition from traditional to westernized lifestyles (Smits et al., 2017; Fragiadakis et al., 2019). In turn, the abundance of potential pathogenic species such as *Acinetobacter* and *Pseudomonas* exhibited the opposite pattern. Interestingly, a recent study has reported that *Pseudomonas aeruginosa*, an opportunistic pathogen from CeD patients, may have proteolytic activity and could trigger a severe intestinal inflammation synergistically with gluten in experimental CeD models (Caminero et al., 2019). All in all, our study provides new insights into the human milk bacterial profile and its potential role in CeD onset in children in a prospective study for the first time. This shows it could serve as a vehicle for vertical transmission of protective or harmful bacteria, whose pathogenic role should be investigated further.

Limitations of our study include the low sample size, the sample heterogeneity (country origin) and the lack of records of relevant clinical data recently demonstrated to impact the hMM such as milk expression method (Moossavi et al., 2019). Moreover, milk sampling for analysis (9 months after birth) could have been completed at an earlier point, given that infants’ gut microbiota modulation by breastfeeding also starts earlier. However, this analysis could only be done at 9 months because: earlier samples were processed for macronutrient and endocrine profiling (Grunewald et al., 2019), all available milk sample from mothers included in this pilot study were uniform at this time point, and because all were still breastfeeding their children, together with the introduction of complementary food. Accordingly, we assumed that hMM would still act as a modulator of the infants’ gut microbiota. In spite of the limitations, this pilot study holds promise to identify new factors that could affect hMM and impact on CeD onset in larger cohorts and, thus, provide a more comprehensive understanding of moderators and mediators of the disease etiology. Such a robust design would also be necessary to clarify if the hMM profiles could be informative to trace short-term lifestyles changes in Western societies.

## Data Availability Statement

The datasets generated for this study can be found in the MG-RAST server (Meyer et al., 2008), accession number mgp88214.

## Ethics Statement

The studies involving human participants were reviewed and approved by the Medical University of Warsaw, Hospital La Paz, Institut d’Investigació Sanitària Pere Virgili, Hospital La Fe, Heim Pál Children’s Hospital, Dr. von Hauner Children’s Hospital, University of Warmia and Mazury, and Leiden University Medical Center ethics committees. The patients/participants provided their written informed consent to participate in this study.

## Author Contributions

MM and YS designed and coordinated the study. HS, MP-L, IP, GC, MN, CR-K, IK-S, SK, and CM recruited participants in their respective clinical centers and recorded samples-associated metadata. AB-P and MO processed human milk samples and performed amplicon sequencing analyses. AB-P executed massive DNA data analysis. AB-P, MO, MM, and YS drafted the manuscript. All authors contributed to the critical review of the manuscript and approved the final version submitted to this journal.

## Conflict of Interest

The authors declare that the research was conducted in the absence of any commercial or financial relationships that could be construed as a potential conflict of interest.

## Footnotes

1 www.preventcd.com

2 http://cran.r-project.org

## Supplementary Material

FIGURE S1 Principal Coordinate Analysis (PCoA) of samples and negative controls. Bray–Curtis dissimilarity index and distance between samples and negative controls was evaluated with OTU abundance information through multidimensional analysis. Controls (turquoise filled circles), cases/celiac disease (CeD, red filled circles), and negative controls (black filled circles) are represented in this scatter plot, containing information of two main principal coordinates explaining the largest variation among samples.

FIGURE S2 Beta diversity analysis of the human milk microbiota. Multivariate analysis (Principal Coordinate Analysis –PCoA) of OTUs associated with the origin country **(A)** and offspring birth year **(B)**. Distribution of PC1 and PC2 values as a function of the both covariates are shown upon unweighted and weighted unifrac metrics, respectively. Permanova test and *p*-values are shown accordingly. Color legend is shown below respective scatter plots.

FIGURE S3 OTUs most significantly associated with offspring birth year. The OTUs showing the extreme variation between groups (*p* < 0.05) according to LMM analysis were taxonomically identified using the SINA aligner (Pruesse et al., 2012) and SILVA database (Quast et al., 2013). When taxonomic assignment at genus level reached ≥99% sequence identity, identification at the species level using a BlastN-based search against the NCBI 16S rRNA gene non-redundant database (https://blast.ncbi.nlm.nih.gov/Blast.cgi) was completed. The distribution of offspring delivery dates between 2007 and 2010 is presented in the plot at the bottom. The OTUs negatively associated (yearly time-course decrease, in light blue) and positively associated (yearly time-course increase, light red), together with respective taxonomic identification are shown at the top. The association slope is directly correlated to the inter-group differences detected by LMM analysis and the respective *p*-values are shown within square brackets. The abundance (Log) in terms of log_10_ normalized DNA reads counts and prevalence (Prev) discriminated per birth year subgroups are disclosed, respectively.
