# Supplementary figures and images for: Breast-Milk Microbiota Linked to Celiac Disease Development in Children: A Pilot Study From the PreventCD Cohort
Source: Front Microbiol. 2020 Jun 23;11:1335. doi: 10.3389/fmicb.2020.01335 (PMC7324710; doi:10.3389/fmicb.2020.01335)

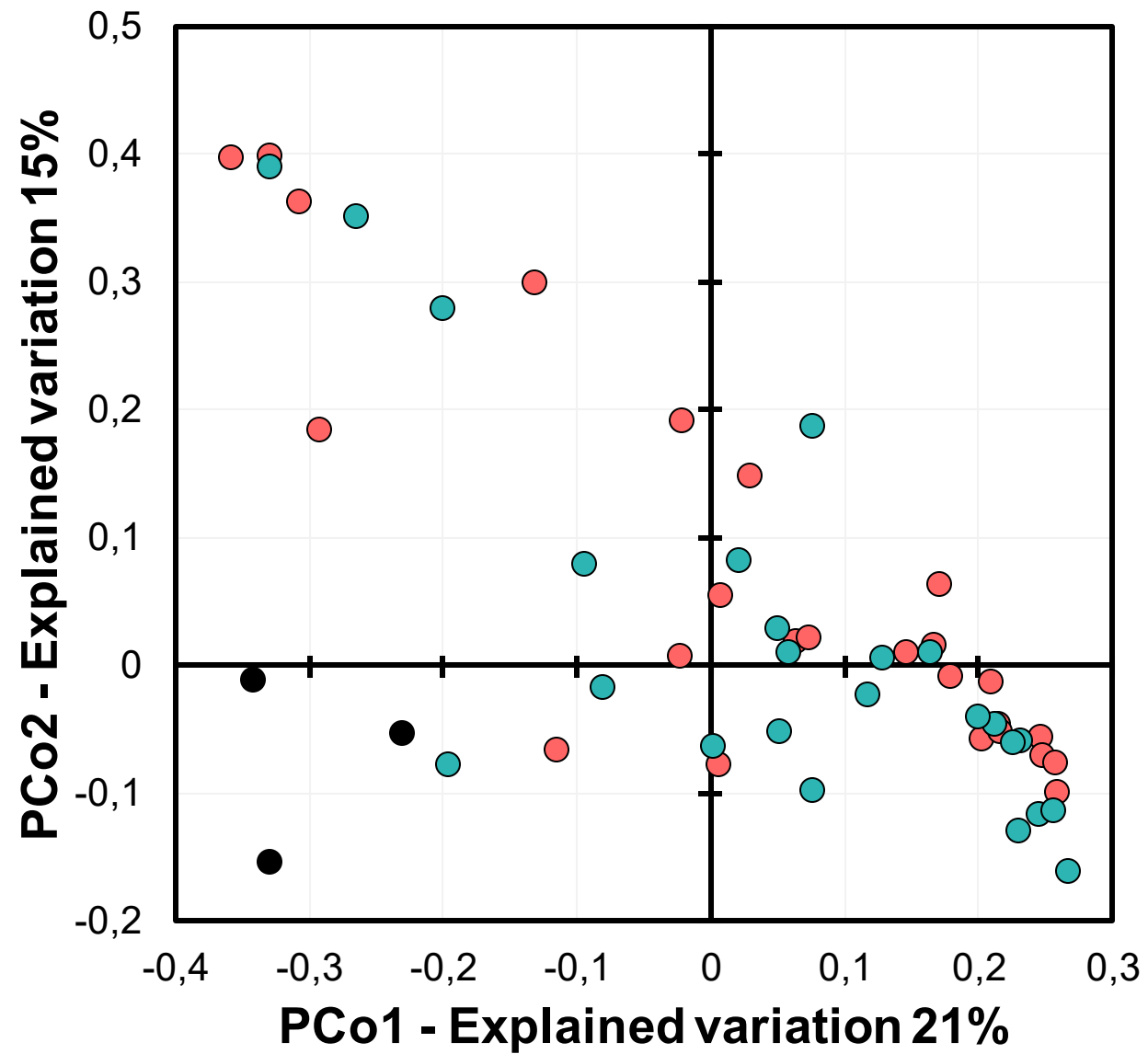

Supplement: FIGURE S1 — Principal Coordinate Analysis (PCoA) of samples and negative controls. Bray–Curtis dissimilarity index and distance between samples and negative controls was evaluated with OTU abundance information through multidimensional analysis. Controls (turquoise filled circles), cases/celiac disease (CeD, red filled circles), and negative controls (black filled circles) are represented in this scatter plot, containing information of two main principal coordinates explaining the largest variation among samples. [file Data_Sheet_1.PDF]

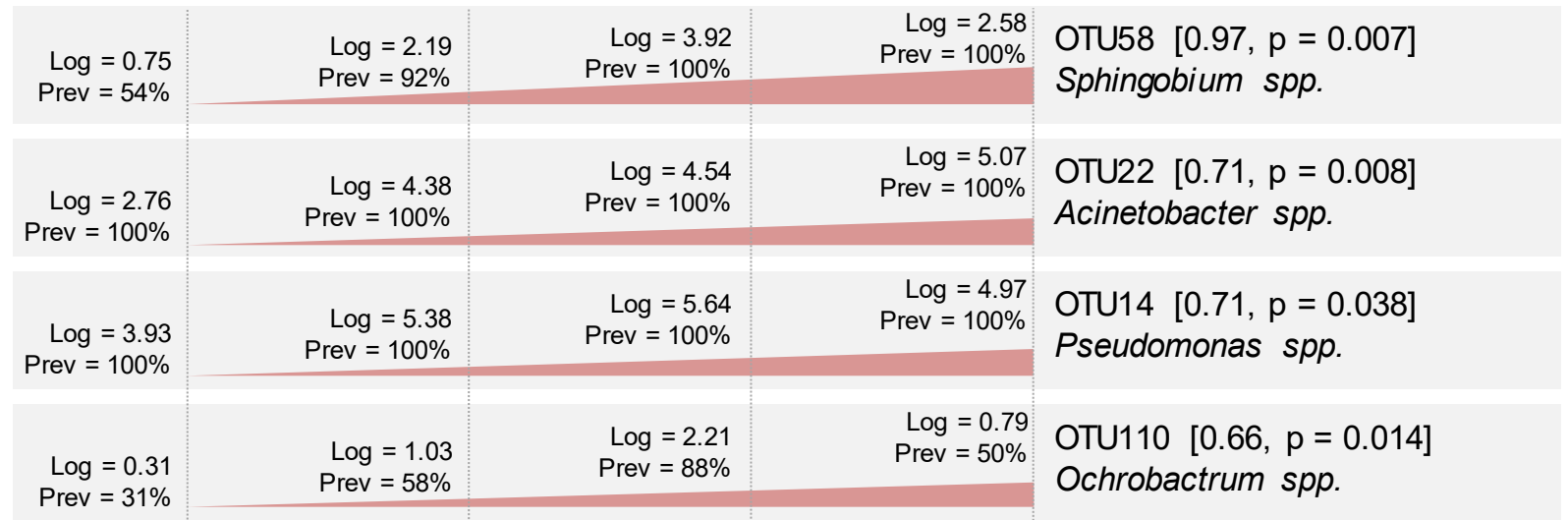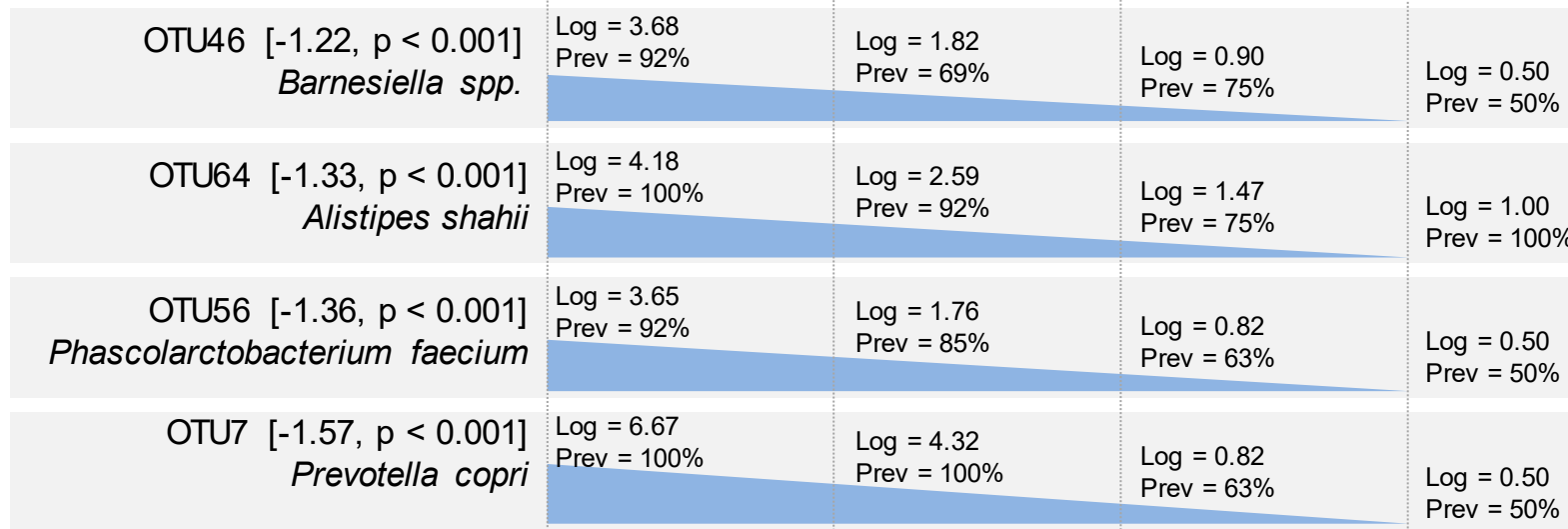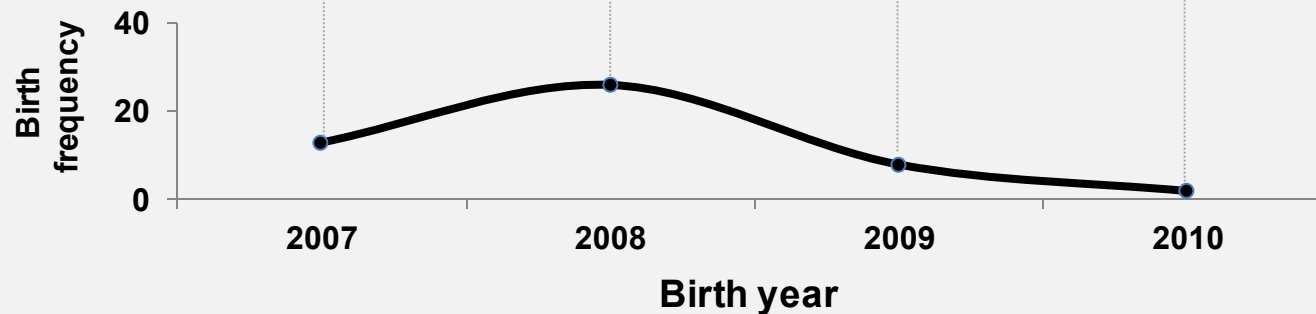

Supplement: FIGURE S3 — OTUs most significantly associated with offspring birth year. The OTUs showing the extreme variation between groups (p < 0.05) according to LMM analysis were taxonomically identified using the SINA aligner (Pruesse et al., 2012) and SILVA database (Quast et al., 2013). When taxonomic assignment at genus level reached ≥99% sequence identity, identification at the species level using a BlastN-based search against the NCBI 16S rRNA gene non-redundant database (https://blast.ncbi.nlm.nih.gov/Blast.cgi) was completed. The distribution of offspring delivery dates between 2007 and 2010 is presented in the plot at the bottom. The OTUs negatively associated (yearly time-course decrease, in light blue) and positively associated (yearly time-course increase, light red), together with respective taxonomic identification are shown at the top. The association slope is directly correlated to the inter-group differences detected by LMM analysis and the respective p-values are shown within square brackets. The abundance (Log) in terms of log10 normalized DNA reads counts and prevalence (Prev) discriminated per birth year subgroups are disclosed, respectively. [file Data_Sheet_3.PDF]
