# Supplementary material for: Breast-Milk Microbiota Linked to Celiac Disease Development in Children: A Pilot Study From the PreventCD Cohort
Source: Front Microbiol. 2020 Jun 23;11:1335. doi: 10.3389/fmicb.2020.01335 (PMC7324710; doi:10.3389/fmicb.2020.01335)
Supplement: FIGURE S2 — Beta diversity analysis of the human milk microbiota. Multivariate analysis (Principal Coordinate Analysis –PCoA) of OTUs associated with the origin country (A) and offspring birth year (B). Distribution of PC1 and PC2 values as a function of the both covariates are shown upon unweighted and weighted unifrac metrics, respectively. Permanova test and p-values are shown accordingly. Color legend is shown below respective scatter plots. [file Data_Sheet_2.PDF]

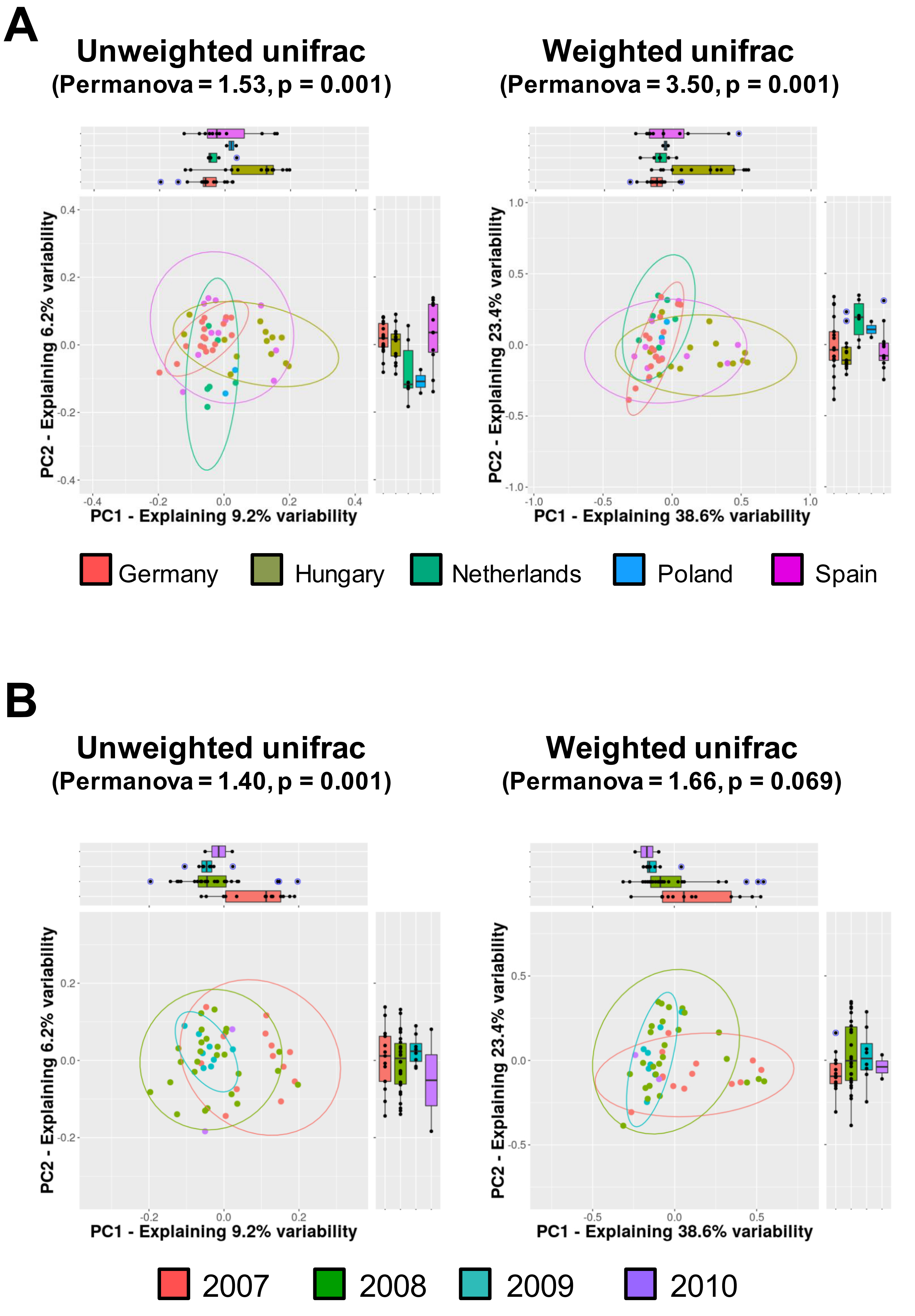
A
Unweighted unifrac
(Permanova = 1.53, p = 0.001)
PC2 - Explaining 6.2% variability
PC1 - Explaining 9.2% variability
Weighted unifrac
(Permanova = 3.50, p = 0.001)
PC2 - Explaining 23.4% variability
PC1 - Explaining 38.6% variability
Germany
Hungary
Netherlands
Poland
Spain
B
Unweighted unifrac
(Permanova = 1.40, p = 0.001)
PC2 - Explaining 6.2% variability
PC1 - Explaining 9.2% variability
Weighted unifrac
(Permanova = 1.66, p = 0.069)
PC2 - Explaining 23.4% variability
PC1 - Explaining 38.6% variability
2007
2008
2009
2010
